# The Influence of Solid and Liquid Systems In Vitro on the Growth and Biosynthetic Characteristics of Microshoot Culture of *Spiraea betulifolia* ssp. *aemiliana*

**DOI:** 10.3390/ijms24032362

**Published:** 2023-01-25

**Authors:** Tatiana V. Zheleznichenko, Dinara S. Muraseva, Andrey S. Erst, Alexander A. Kuznetsov, Maxim S. Kulikovskiy, Vera A. Kostikova

**Affiliations:** 1 Central Siberian Botanical Garden, Siberian Branch of Russian Academy of Sciences (CSBG SB RAS), Novosibirsk 630090, Russia; 2 Department of Natural Sciences, Section of Molecular Biology and Biotechnology, Novosibirsk State University, Novosibirsk 630090, Russia; 3 Laboratory Herbarium (TK), Tomsk State University, Tomsk 634050, Russia; 4 K.A. Timiryazev Institute of Plant Physiology, Russian Academy of Sciences (IPP-RAS), Moscow 127276, Russia

**Keywords:** *Spiraea betulifolia* ssp. *aemiliana*, in vitro culture, flavonoid, phenol carboxylic acid, solid culture medium, liquid culture medium

## Abstract

The paper focuses on the growth dynamics and biosynthetic characteristics of the microshoot culture of *Spiraea betulifolia* ssp. *aemiliana* obtained in vitro in agar-solidified and liquid media. Microshoots cultured in either type of media showed similar growth dynamics. The most active culture growth was observed from day 35 to day 60. A comparative analysis of the contents of flavonoids and phenol carboxylic acids showed a higher level of phenol carboxylic acids (5.3–6.84%) and a stronger 1,1-diphenyl-2-picrylhydrazyl (DPPH) radical–scavenging activity (half-maximal inhibitory concentration: 341 µg/mL) in *S. betulifolia* ssp. *aemiliana* microshoots grown in the liquid medium compared to the microshoots cultured in the solid medium. The flavonoid content of the cultured microshoot did not depend on the consistency of the medium. High-performance liquid chromatography (HPLC) was employed to study the profile and levels of phenolic compounds in microshoots, intact plants, and ex vitro–acclimated *S. betulifolia* ssp. *aemiliana* plants. The concentration of kaempferol glycosides was found to be higher in microshoots (1.33% in the solid medium, 1.06% in the liquid medium) compared to intact plants and ex vitro–acclimated plants. Thus, the microshoots of *S. betulifolia* ssp. *aemiliana* cultured in the liquid medium rapidly increase their biomass and are an inexpensive promising source of biologically active antioxidant substances, mainly phenol carboxylic acids and kaempferol glycosides.

## 1. Introduction

Plants are currently the only source of some biologically active substances because the chemical synthesis of these substances is complicated and economically infeasible [1]. At present, numerous medicinal plants are endangered due to the massive procurement of raw materials for the pharmaceutical industry [2]. Therefore, alternative methods are needed to obtain medicinal raw materials, e.g., an in vitro method for the production of secondary plant metabolites. Until recently, plant tissue and organ culture techniques have mainly been used to propagate rare and endangered plants and plant species that are difficult to propagate by conventional methods. On the other hand, in vitro approaches are gaining popularity; they are widely utilized for the commercial propagation of ornamental, fruit, berry, and medicinal plants [3,4,5]. In vitro approaches provide high-quality planting material or standardized medicinal raw material within a short period regardless of the season and geographical or environmental factors. Suspension culture is most often used to produce secondary metabolites owing to relatively fast growth and the feasibility of culturing in bioreactors. An example of successful in vitro biosynthesis of medicinal-plant secondary metabolites is the production of solasodine from callus cultures of *Solanum elaeagnifolium* [6]. Effective biosynthesis of indole alkaloids has been achieved in the suspension culture of *Catharanthus roseus* [7]. Cephaeline and emetine have been isolated from callus cultures of *Cephaelis ipecacuanha* [8].

Nonetheless, some biochemical processes in higher plants can occur only in certain organs or during certain developmental stages. Therefore, the accumulation of some metabolites may depend on the presence of certain types of cells or organelles and on the expression and regulation of certain biosynthetic genes [9]. For this reason, scientists attempt to develop technologies for culturing organs, including somatic embryos, roots, or shoots. For example, the productive biosynthesis of pyrrolizidine alkaloids is observed in the adventitious root culture of *Senecio* sp. [10]. It has also been revealed that the accumulation of azadirachtin and nimbin in the microshoots and roots of *Azadirachta indica* cultured in vitro is higher than that recorded in native plants grown in fields [11], indicating a good potential for this method.

The commercial in vitro culture of differentiated organs is currently hardly feasible. One of the major reasons limiting industrial-scale production is the use of mainly agar-solidified media in most of the described clonal micropropagation protocols, and this approach increases production costs. Therefore, one of the current challenges is to reduce costs and increase production efficiency. The application of liquid culture media to clonal micropropagation is one strategy to reduce costs.

Our previous studies [12,13] were the first to present a protocol developed for clonal micropropagation of the ornamental shrub *Spiraea betulifolia* ssp. *aemiliana* (C.K. Schneid.) H. Hara (syn. *S. aemiliana* C.K. Schneid.) in Murashige and Skoog (MS) agar media [14] and documented the effective biosynthesis of some phenolic compounds as compared to intact plants.

*S. betulifolia* ssp. *aemiliana* is a representative of the section *Calospira* C. Coch and grows only in the insular part of Russia (Sakhalin Island, the Kuril Islands) and in Japan. This shrub is of interest because the *Spiraea* species is able to accumulate biologically active substances, including phenolic compounds, particularly flavonoids and phenol carboxylic acids [15,16,17]. The presence of these substances in leaves and inflorescences of *Spiraea* species explains the various functional effects of these plants, for example, antioxidant, antitumor, antiviral, anti-inflammatory, anti–α-amylase, antiadipogenic, and antilipogenic [16,18,19,20,21]. Designing in vitro culture systems for *S. betulifolia* ssp. *aemiliana* is of high relevance due to the practically important qualities of the plants and the limited availability of this raw material in natural habitats. In addition, a comparative analysis of the dynamics of accumulation of biologically active substances in in vitro–cultured plants should reveal the potential of various types of culture to generate target metabolites.

The aims of the study were (i) modification of a protocol for clonal micropropagation of *S. betulifolia* ssp. *aemiliana* in a liquid medium; (ii) a comparison of the biomass productivity dynamics of the obtained microshoot cultures in agar-solidified and liquid media; and (iii) a comparative analysis of the content of biologically active substances and antioxidant activity of plants in vitro and in vivo.

## 2. Results and Discussion

### 2.1. Microshoot Propagation in Liquid and Solid Media

Feasibility of culturing *S. betulifolia* ssp. *aemiliana* in the MS liquid medium was tested first. The results showed that for the successful growth of *S. betulifolia* ssp. *aemiliana* in the liquid medium, the plant material must first be grown in a solid medium for no less than eight passages after the introduction of vegetative buds into the in vitro culture. Shorter in vitro culture of the studied *Spiraea* species in the solid medium and transfer of the culture to the liquid medium caused vitrification of the plant material, hampered its growth, and led to culture necrosis. Our experiments also indicated that during cultivation in the liquid medium, the ratio of the medium volume to the inoculum (explant) mass should be monitored. For example, culture-based production of 100 mg of *S. betulifolia* ssp. *aemiliana* raw biomass requires a medium volume of ~25 mL.

Liquid media significantly reduce the costs of in vitro culturing, yet they are rarely used for clonal micropropagation because deep immersion of the plant material into a liquid medium and the absence of agar or its decreased content causes vitrification of the plant material [22,23,24]. A number of papers suggest that not all plant species are prone to vitrification [25,26]. For instance, it has been found that culturing in a liquid medium succeeds if plant material is not immersed in the bulk of the liquid medium but rather floats on the surface [27]. This could be the reason for our successful culturing of *S. betulifolia* ssp. *aemiliana* in the liquid medium.

**Figure 1:**
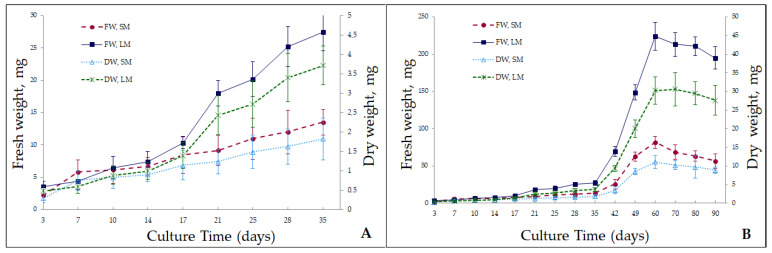
The growth curve for *S. betulifolia* ssp. *aemiliana* microshoots in hormone-free MS solid (agar) and liquid media for (**A**) 35-day (**B**) and 90-day cultivation. FW SM: fresh weight for the solid medium; FW LM: fresh weight for the liquid medium; DW SM: dry weight for the solid medium; DW LM: dry weight for the liquid medium.

Growth characteristics of microshoots of the analyzed *Spiraea* species were investigated in hormone-free MS solid and liquid media for 90 days in terms of wet and air-dry biomass (Figure 1). Growth patterns of the biomass were similar between the solid and liquid media. The graph shows approximately six growth phases. The first few days of culturing are the lag phase, when biomass shows no visible increase and the cultured plant adapts to the new conditions. For the plant in the solid medium, this interval was shorter and amounted to approximately 3–5 days; on day 7, the biomass increased 2.5-fold compared to the initial value. For the plant in the liquid medium, the lag phase was longer: 7–10 days. Biomass increased slightly, but the growth index was less than two. Next, the biomass grew stepwise in both types of media, and three growth phases could be distinguished. The first growth phase (G-I) lasted for up to 17 days. During this period, the culture did not exhibit active growth; the biomass grew no more than 2-fold. On day 17, the growth index reached 3.9 for the solid medium and 2.9 for the liquid medium. It was also noted that during the first 17 days of cultivation, biomass growth was more active in the solid medium than in the liquid one. The second growth phase (G-II) lasted from day 17 to day 35; it was designated as pre-exponential. During this phase, the biomass grew approximately 4–5-fold. In the liquid medium, it grew more actively than in the solid one. On day 35, the growth index was 6.25 in the agar medium and 7.7 in the liquid medium. The period from day 35 to day 60 was the next growth phase (G-III), which could be described as exponential. During this period, the biomass increased dramatically, especially in the liquid medium. On day 60, the growth index was 37.9 in the solid medium and 63.3 in the liquid medium. Culture growth is more efficient in a liquid medium because nutrients become more available to plants owing to the closer contact between the explant and the medium [28,29]. In addition, the harmful effects of toxins are minimized in the liquid culture systems. Metabolites released by explants into the medium can have an inhibitory or toxic effect on further growth and/or development of the cultured plant. In solid media, the released metabolites are located close to the explant, whereas in liquid media, the metabolites get washed out [30]. Apical dominance diminishes significantly, thereby stimulating the induction and growth of numerous axillary buds, thus initiating a large number of microshoots [31].

**Figure 2:**
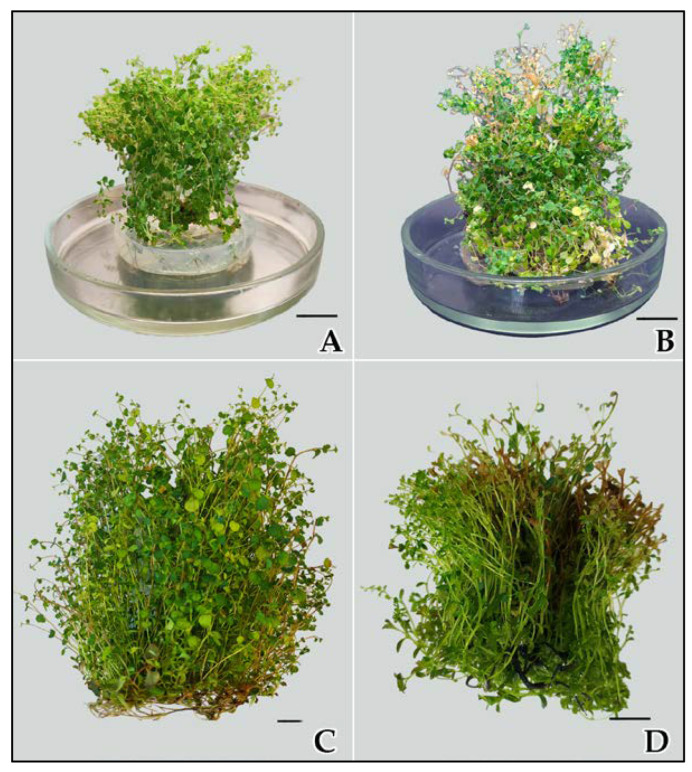
Microshoots of *S. betulifolia* ssp. *aemiliana* after (**A**) 60 days, (**B**) and 90 days of culturing in the agar-solidified medium, and after (**C**) 60 days, (**D**) and 90 days of culturing in the liquid medium. Scale bar: 1 cm.

After that, from day 60, a very short phase of stationary growth of the culture could be observed in both types of media. It lasted no more than 10 days and gradually transitioned into the degradation phase. Starting from day 60, microshoot biomass quality declined sharply, especially in the liquid medium. Chlorosis and leaf fall occurred, and by day 90, microshoot necrosis was registered (Figure 2). The sharp culture degradation can be attributed to the faster depletion of mineral nutrients in the liquid medium than in the solid one and probably to the release of toxic metabolites into the culture liquid.

Thus, the microshoots of *S. betulifolia* ssp. *aemiliana* in vitro–cultured in solid and liquid systems showed that the period of initial adaptation of the cultured plant to the liquid medium is slightly longer (than that to the solid medium), and then, biomass rapidly grows, and finally, the growth is sharply inhibited. In the solid medium, the growth of the *S. betulifolia* ssp. *aemiliana* culture was not active, yet, the cultivation could be longer. Despite the more active growth of *S. betulifolia* ssp. *aemiliana* in liquid media, it is known that standardized media are not always suitable for the optimal growth of many wild plant species. In this regard, in the future, it is necessary to study optimal concentrations of mineral salts in the nutrient medium, as has already been done for hazelnut and strawberry [32,33]. This work may improve the growth and biosynthetic characteristics of plant culture.

### 2.2. Contents of Flavonoids and Phenol Carboxylic Acids in S. Betulifolia Ssp. Aemiliana In Vitro and In Vivo

**Figure 3:**
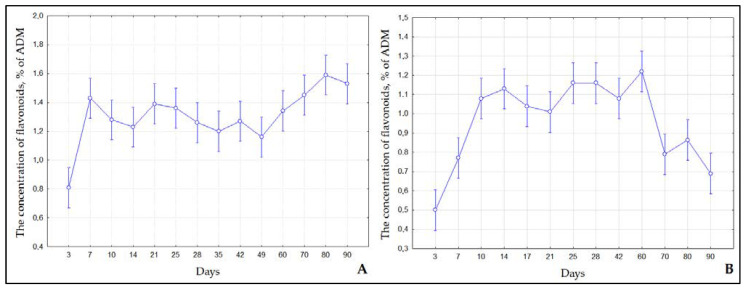
The concentration of flavonoids in microshoots of *S. betulifolia* ssp. *aemiliana* cultured in (**A**) agar-solidified (**B**) and liquid media.

We analyzed the dynamics of the accumulation of flavonoids and phenol carboxylic acids in microshoots of *S. betulifolia* ssp. *aemiliana* cultured in solid (MshS) and liquid (MshL) media for 90 days. An analysis of water-ethanol MshS extracts showed that the flavonoid level was the lowest (0.81%) on day 3 (lag phase) (Figure 3a). From day 7 to day 90, the flavonoid content of MshS went up and varied within 1.16–1.59%. The flavonoid concentration in microshoots of *S. betulifolia* ssp. *aemiliana* cultured in the liquid medium differed from that in the solid medium. In the liquid medium, the flavonoid content was low on days 3 (0.5%) to 7 (0.77%). Then, during the period of active growth from day 10 to day 60, the flavonoid content of microshoots reached a plateau and attained its maximum (1.01% to 1.22%). During the degradation phase, the flavonoid level decreased, and from day 70 to day 90, it ranged from 0.69% to 0.87%. Concentrations of phenolic compounds, in particular flavonoids, changed in the *S. betulifolia* ssp. *aemiliana* microshoot culture since phenolic compounds are metabolically active plant compounds [34]; they are characterized by relatively rapid turnover and degradation. For example, phenols may be involved in various biosynthetic processes, undergo catabolism with subsequent conversion into primary metabolites, or participate in oxidative polymerization reactions, which cause the formation of insoluble structures of high molecular weight [34]. Therefore, it is worthwhile to identify the stage when culture productivity reaches its maximum.

**Figure 4:**
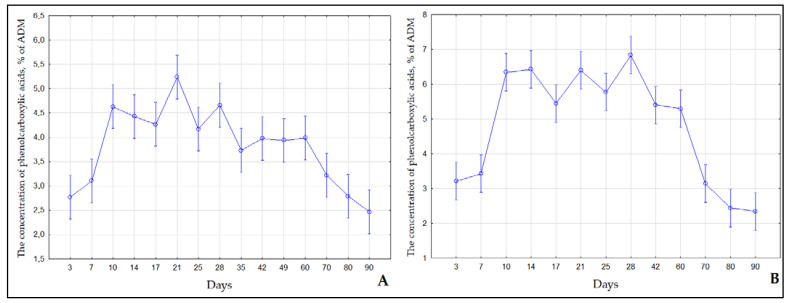
The concentration of phenol carboxylic acids in microshoots of *S. betulifolia* ssp. *aemiliana* cultured in (**A**) agar-solidified (**B**) and liquid media.

The patterns of accumulation of phenol carboxylic acids during the cultivation of *S. betulifolia* ssp. *aemiliana* were similar between the different types of media (Figure 4). From day 3 to day 7, the phenol carboxylic acids content of MshS and MshL was low, consistently with the lag phase of biomass growth, when the active biosynthesis of secondary metabolites does not proceed. From day 10 to day 60, during the active growth phase (pre-exponential and exponential), the content of phenol carboxylic acids rose sharply. The maximum content of phenol carboxylic acids in MshS varied within 3.73–5.24%, whereas in MshL, the maximum content of phenol carboxylic acids reached 5.3–6.84%, which is slightly higher in comparison with MshS. On day 70 (degradation phase), the phenol carboxylic acids content of the plant material started to diminish gradually. This is probably due to the involvement of phenol carboxylic acids in the active formation of plant cell walls or in microshoot lignification, where these compounds serve as the main structural lignin units [35].

Plant cell and tissue cultures, including both dedifferentiated culture (callus and suspension culture) and differentiated one (microshoot or root culture), can serve as alternative producers of phenolic compounds [36,37,38]. The biosynthetic properties of *S. betulifolia* ssp. *aemiliana* cultured in the liquid medium should be improved further to obtain a higher content of phenol carboxylic acids because they are a key class of polyphenols and are widely used in the human diet. The daily intake of phenol carboxylic acids for an individual is ~200 mg or more [39]. Phenol carboxylic acids are readily absorbed through the walls of the gastrointestinal tract and are beneficial to human health owing to their potential antioxidant properties. They prevent cell damage caused by free-radical oxidation reactions [40]. Regular consumption of phenol carboxylic acids enhances the anti-inflammatory abilities of the human immune system [41]. Industry demand for phenol carboxylic acids is high because they work as precursors to other important bioactive molecules that are needed in the medical, cosmetic, and food industries. The growing need for phenol carboxylic acids is also associated with environmental conservation because they are metabolized by natural microorganisms [42].

A comparison of the content of biologically active substances in water-ethanol leaf extracts between intact plants (IPs) and ex vitro–acclimated plants (APs) showed that the flavonoid content of these plants exceeds that registered in MshS and MshL extracts (Table 1). The phenol carboxylic acids level in IPs and APs was equal to that observed in MshL. It should be noted that the flavonoid contents were virtually equal between IPs and APs, and the phenol carboxylic acids content of APs slightly exceeded that of IPs (Table 1).

The practical potential of *S. betulifolia* ssp. *aemiliana* is related to its ability to accumulate flavonoids, especially flavonols: hyperoside, isoquercitrin, rutin, quercetin, kaempferol, and astragalin. The level of flavonols was determined when the content of phenolic compounds was assayed in the leaves of plants from natural and introduced coenopopulations [43]. The profile and levels of flavonoids and phenol carboxylic acids were analyzed via High-performance liquid chromatography (HPLC) of IPs and APs leaf extracts as well as MshS and MshL collected on day 60. The set of detected phenolic compounds in MshS was not diverse (10 compounds). The phenolic-compound profile of MshL extracts was more diverse (~16 compounds). A total of 26 phenolic compounds were identified in IPs and APs leaf extracts.

IPs and APs leaf extracts were found to contain three phenol carboxylic acids (chlorogenic, *p*-coumaric, and cinnamic) and seven flavonols (taxifolin, hyperoside, isoquercitrin, rutin, astragalin, quercetin, and kaempferol; Table 2 and Table 3). The compounds were identified using both UV spectra and a comparative analysis of their chromatographic retention times and those of standard samples. Other phenolic compounds were not identifiable by the above method. Their UV spectra were detected online by chromatography. On the basis of the spectral characteristics of the unidentified phenolic compounds, they were classified as flavonols (λ_max_ = 250–270 and 350–390 nm), phenol carboxylic acids (oxybenzoic (λ_max_ = 235–270 and 290–305 nm) or oxycinnamic acids (λ_max_ = 230–240 and 290–320 nm)), or flavones (λ_max_ = 250–270 and 210–350 nm). Chlorogenic and *p*-coumaric acids, quercetin, and kaempferol were found in MshS, whereas taxifolin and astragalin were identified in MshL (Table 2 and Table 3).

In previous work, the concentrations of chlorogenic acid, kaempferol, and phenolic acid 4 were reported to be 1.5–2-fold higher in *S. betulifolia* ssp. *aemiliana* cultured in vitro (depending on the culture stage in an agar-solidified medium) than in IPs, while the levels of hyperoside, astragalin, and quercetin were higher in the IPs [13]. Table 2 and Table 3 present the detected phenolic compounds and the undetected substances whose concentrations in water-ethanol leaf and microshoot extracts of *S. betulifolia* ssp. *aemiliana* were >1 mg/g. Major phenolic compounds in IPs and APs leaf extracts were phenolic acid 4, hyperoside, flavone 5, and quercetin; in microshoots, these were phenolic acid 4, chlorogenic acid, quercetin, and kaempferol. In microshoot extracts, the content of kaempferol (0.25 mg/g in MshL, 0.31 mg/g in MshS) was 2–2.6-fold higher compared to leaf extracts (0.12 mg/g in IPs and 0.14 mg/g in APs). Contents of other substances identified in microshoots of *S. betulifolia* ssp. *aemiliana* were similar to those in IPs and APs (chlorogenic acid) or lower (*p*-coumaric acid, acid 4, taxifolin, flavone 5, astragalin, and quercetin).

In hydrolysates of the assayed *Spiraea* extracts (after hydrolysis with hydrochloric acid), aglycones were identified: quercetin and kaempferol (Table 3). When the content of aglycones was recalculated relative to the corresponding glycoside by means of conversion factors available in the literature, quercetin glycosides were found to dominate in the studied samples. The level of quercetin glycosides in IPs (9.23 mg/g) and APs (8.83 mg/g) leaf extracts was 9-fold higher than that in microshoot extracts cultured in solid (1.38 mg/g) and liquid (1.24 mg /g) media. The elevated content of quercetin glycosides in IPs and APs leaves is most likely due to the effect of UV radiation on open-ground plants. In many cases, quercetin glycosides with ortho-dihydroxyl groups on the B ring are effective against UV radiation, and plant leaves synthesize more quercetin and its derivatives under UV-B radiation [44,45]. In IPs and APs, the concentration of kaempferol glycosides was 10–11-fold lower than the concentration of quercetin glycosides (Table 3). Contents of quercetin glycosides and kaempferol glycosides were virtually equal in MshS as well as in MshL. The level of kaempferol glycosides was higher in microshoots (in vitro) than in IPs and APs. Additionally, the content of kaempferol glycosides in MshS (1.33 mg/g) was slightly higher than that in MshL (1.06 mg/g). Among plants, kaempferol and its glycosides are widespread polyphenolic flavonoids of high pharmacological and nutraceutical potential. To date, more than 350 kaempferol derivatives have been identified in plants, but their role in plants has been poorly studied [46]. It is known that kaempferol glycosides can act as copigments and attractants for pollinators [47]. It has been demonstrated that these compounds can act as UV shields for plants [46,48,49]. Moreover, the antioxidant activity of kaempferol derivatives in plant cells has been confirmed [50]. Kaempferol is an effective phytoalexin against the crop pathogen *Verticillium albo-atrum* [51]. El-Gammal and Mansour [52] have revealed that kaempferol exerts antimicrobial action against various bacterial and fungal strains evaluated as test organisms. On the other hand, it induces the proliferation of nitrogen-fixing bacteria [53]. Furthermore, kaempferol and its glycosides affect seed formation [54] and plant development [55]. We believe that kaempferol and its derivatives play an important role in the protection and development of microshoots during in vitro culture of *S. betulifolia* ssp. *aemiliana*.

Kaempferol and its glycosides are often isolated from plants used in folk medicine owing to their antimicrobial properties. Numerous research articles suggest that plants containing kaempferol, its glycosides, or kaempferol, have antibacterial, antiviral, antifungal, and antiprotozoal activities [46]. It has been reported that these compounds can have antitumor, anti-inflammatory, and antioxidant effects and possess hypoglycemic and antidepressant properties [56,57]. Recent studies point to the potential utility of kaempferol and its derivatives for the treatment and prevention of COVID-19 and for the elimination of its complications [58,59]. The therapeutic value of *S. betulifolia* ssp. *aemiliana* can be increased in vitro with the help of various elicitors, mainly kaempferol and its derivatives.

### 2.3. Antioxidant Activity of S. Betulifolia Ssp. Aemiliana In Vitro and In Vivo

**Figure 5:**
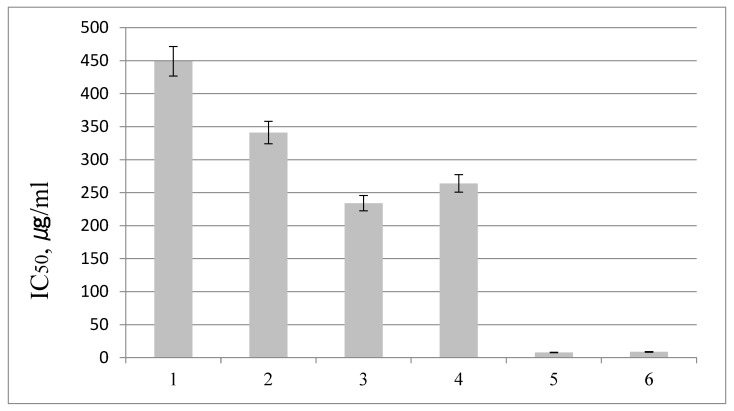
*DPPH radical*–scavenging *activity of S. betulifolia* ssp. *aemiliana* extracts. IC_50_, µg/mL, is the antioxidant concentration that causes 50% inactivation of the DPPH radical. 1: MshS, day 60; 2: MshL, day 60; 3: IPs; 4. APs; 5: trolox; 6: ascorbic acid.

We tested the antioxidant activity of *S. betulifolia* ssp. *aemiliana* extracts from microshoots collected on day 60 (Figure 5). A comparative analysis was performed on the antioxidant activities of IPs and APs leaf extracts. The MshL extract (half-maximal inhibitory concentration [IC_50_] = 341 µg/mL) proved to be more active than the MshS extract (IC_50_ = 449 µg/mL), consistently with the higher content of phenol carboxylic acids in the MshL extract. IPs (IC_50_ = 234 µg/mL) and APs (IC_50_ = 264 µg/mL) extracts manifested a stronger 1,1-diphenyl-2-picrylhydrazyl (DPPH) radical–scavenging activity compared to MshS and MshL extracts, most likely because of the higher concentration of flavonoids, mainly quercetin glycosides. The *Spiraea* extracts were found to have a significantly lower antioxidant activity relative to 6-hydroxy-2,5,7,8-tetramethylchroman-2-carboxylic acid (trolox) and ascorbic acid.

## 3. Materials and Methods

### 3.1. Plant Material

The aseptic culture of *S. betulifolia* ssp. *aemiliana* obtained in the Laboratory of Biotechnology in the CSBG SB RAS (Novosibirsk) was used in this study [12,13]. The explants were axillary buds of a generative plant introduced into the experimental field of the Laboratory of Phytochemistry (CSBG SB RAS, Russia) from Kunashir Island (the caldera of Golovin’s volcano). Microshoots were incubated under a 16 h photoperiod at 40 μmol m^−2^ s^−1^ light intensity provided by cool white fluorescent lamps at 23 ± 2 °C in the MS [14] solid medium (supplemented with 0.6% of agar; PanReac, Barcelona, Spain) containing 3% of sucrose (Shostka Chemica l Reagents Plant, Shostka, Ukraine). Micropropagation of *S. betulifolia* ssp. *aemiliana* was carried out in the medium containing growth regulators: 5 µM 6-benzylaminopurine (Sigma-Aldrich, St. Louis, MO, USA) and 1 µM α-naphthylacetic acid (Sigma-Aldrich, St. Louis, MO, USA); elongation was performed in hormone-free media of the same mineral composition. Passage duration was 4–6 weeks.

### 3.2. Examination of Culture Growth Dynamics in Solid and Liquid Media

Prior to the assessment of the in vitro growth characteristics of *S. betulifolia* ssp. *aemiliana*, microshoots were cultured for three passages in the hormone-free MS solid or liquid medium (without agar); the passage duration was 45 days. Culture growth dynamics were studied during the fourth passage. Explants (microshoots with 3–5 nodes) were pre-weighed under aseptic conditions. In the solid medium, 5–6 explants were cultured in a jar. In the liquid medium, each explant was cultivated individually in 50 mL Erlenmeyer flasks on a shaker tray (ELMI, S-3-02 L, Riga Latvia) at 100 rpm, while the volume of the medium in the flask did not exceed 5 mL. Samples were taken on days 3, 7, 10, 14, 17, 21, 25, 28, 35, 42, 49, 60, 70, 80, and 90 and weighed. Growth curves were constructed for wet and air-dry weights of the biomass. The air-dry weight was measured after drying the material at room temperature in the shade. For each data point in the graph, 10–20 explants were used. The growth index was calculated by means of the formula [60]:

Growth index = X_max_/X_o_,

where X_max_ is the final biomass weight, and X_0_ is the initial biomass weight. *S. betulifolia* ssp. *aemiliana* was cultured in vitro under a 16 h photoperiod at 40 μmol m^−2^ s^−1^ light intensity provided by fluorescent lamps at 23 ± 2 °C in both solid and liquid media.

### 3.3. Plant Material for Phytochemical Assays

Profiles and levels of flavonoids and phenol carboxylic acids, as well as the antioxidant activity of water-ethanol microshoot extracts of *S. betulifolia* ssp. *Aemiliana*, were determined as a function of culture duration in samples cultured in vitro in the hormone-free solid and liquid media.

A comparative analysis was performed based on qualitative and quantitative assays of phenolic compounds and antioxidant activity of water-ethanol leaf extracts of *S. betulifolia* ssp. *aemiliana* IPs introduced into the experimental field of the Laboratory of Phytochemistry in the CSBG SB RAS (Novosibirsk, Russia). The plants were introduced into the experimental field of the CSBG SB RAS from a natural population growing on Kunashir Island (the caldera of Golovin’s volcano) in 2016 (IPs). We investigated leaf extracts of in vitro–propagated plants adapted to ex vitro conditions (leaves of plants ex vitro adapted and acclimated to the experimental field) and planted in 2020 in the experimental field of the Laboratory of Phytochemistry (APs). Plants from the experimental field were collected during the flowering stage (July 1, 2021).

### 3.4. Extract Preparation

To assess the biosynthetic and antioxidant characteristics of *S. betulifolia* ssp. *aemiliana*, the plant material was air-dried completely at room temperature in the shade and weighed. After that, the dry material was shredded into 2–3 mm pieces and blended, and representative samples were chosen. Phenolic compounds were identified and quantified in 70% ethanol-water extracts obtained via extraction on a water bath WB-4MS (BioSan, Riga, Latvia). The extracts were prepared at a 1:500 ratio of the raw material to solvent.

### 3.5. The Total Flavonoid Content

Flavonoids were quantified by the spectrophotometric aluminum chloride technique [61]. Briefly, 0.5 mL of 2% aluminum chloride (Reaktiv, St. Petersburg, Russia) in ethanol (Constanta-Farm M, Moscow, Russia) was mixed with the same volume of the plant extract. After 1 h, absorbance readings at 415 nm against a blank (ethanol) were taken. The optical density of the mixture was measured on an SF-56 spectrophotometer (Lomo, St. Petersburg, Russia). The content of each flavonoid was found based on a graph plotted for rutin (Sigma-Aldrich, St. Louis, MO, USA). The results were expressed in % of absolutely dry mass.

### 3.6. The Total Phenolic Acid Content

The quantitative determination of phenol carboxylic acids was performed by the direct spectrophotometric method because of the maximum absorption of an alcoholic solution of *S. betulifolia* ssp. *aemiliana* extract at 325 nm is equal to that of caffeic acid [19]. The optical density of the extract diluted 5-fold with 96% ethanol was measured on the SF-56 spectrophotometer (Lomo, St. Petersburg, Russia) at 325 nm in a cuvette with 1 cm light path. A reference solution was 96% ethyl alcohol. The concentration was calculated based on a caffeic-acid (Sigma-Aldrich, Taufkirchen, Germany) calibration curve. The results were expressed in % of absolutely dry mass.

### 3.7. Quantitation of Individual Phenolic Compounds via HPLC

This analysis was performed using an Agilent 1200 HPLC system that included a Zorbax SB-C18 column (5 mm, 4.6 × 150 mm) and was equipped with a diode array detector and a ChemStation system for collection and processing of chromatographic data (Agilent Technology, Santa Clara, CA, USA) by van Beek’s method [62] with modifications. Separation was conducted under the following conditions: for 27 min, a gradient from 31% to 33% of methanol (Himmed, Moscow, Russia) acidified with phosphoric acid (Vekton, St. Petersburg, Russia); in the mobile phase, the concentration of methanol in the solution of phosphoric acid (0.1%) was changed from 33% to 46% during 11 min, then from 46% to 56% for the next 12 min, and from 56% to 100% for 4 min (*solvent system I*). The eluent flow rate was 1 mL/min, the column temperature was 26 °C, the sample volume was 10 mL, and the detection was conducted at wavelengths 254, 270, 290, 340, 360, and 370 nm. Quantification of individual compounds in the plant extract samples was conducted by the external standard method [60]. For the detection of phenolic compounds in the plant extracts, standard samples of cinnamic acid (Serva, Heidelberg, Germany), taxifolin (Austrowaren; Austria), chlorogenic and *p-*coumaric acids, quercetin, kaempferol (Sigma-Aldrich, Taufkirchen, Germany), isoquercitrin, rutin, astragalin, and hyperoside (Fluka Chemie AG, Buchs, Switzerland) were employed, which were quantified by the external standard method. The analysis of free aglycones formed after acid hydrolysis of the corresponding flavonoid glycosides was carried out due to the lack of available standard samples and complicated separation conditions. To this end, 0.5 mL of HCl (2 N) (Soyuzkhimprom, Novosibirsk, Russia) was added to 0.5 mL of an extract. The mixture was heated in a boiling water bath for 2 h. A chromatographic analysis was conducted in the gradient elution mode in *solvent system II*: in the mobile phase, the methanol concentration in the aqueous solution of phosphoric acid (0.1%) changed from 45% to 48% in 18 min. Detection was conducted at a wavelength of 370 nm. Concentrations of flavonoid glycosides (glycosides of quercetin, kaempferol) in the plant extract samples were calculated from the levels of free aglycones that formed after the acid hydrolysis. Coefficients from the literature (2.504 for quercetin and 2.588 for kaempferol) were employed to convert the aglycone concentrations to the concentrations of the corresponding glycosides [62]. The results were expressed in mg/g.

### 3.8. Estimation of Antiradical Activity

The free-radical–scavenging capacity of the samples was determined by the DPPH method [63,64] with modifications. For this purpose, a 2 mL aliquot of an extract (dissolved in 70% ethanol to form concentrations in the range of 110–2450 µg/mL) was mixed with 3 mL of a DPPH (TCI, Portland, OR, U.S.A.) solution (62 µg/mL in ethanol). After a 30 min incubation in darkness at room temperature, optical density (A) was measured at 517 nm against a blank sample. Free-radical scavenging activity was calculated as percentage inhibition using the following formula:

I% = (A_blank_ − A_sample_/A_blank_) × 100,

where A_blank_ is the optical density of a control solution (containing all reagents except the tested extracts), and A_sample_ is the optical density of the sample.

The results were expressed in IC_50,_ defined as the concentration of an antioxidant that causes 50% DPPH loss in the DPPH radical–scavenging activity assay. Trolox (Acros Organics, Geel, Belgium) and Ascorbic acid (Reahim, Samara, Russia) solutions (2.5–50.0 µg/mL) were positive controls.

### 3.9. Chemicals

All chemicals were of HPLC or analytical grade.

### 3.10. Statistical Analysis

The data were statistically processed by conventional methods in STATISTICA 6.0 and GraphPad Prism v.6.01 software (GraphPad Software, USA). All the phytochemical experiments were set up with two biological replicates and with three technical replicates per treatment. Multiple comparisons were performed by one-way ANOVA followed by Tukey’s HSD test to evaluate the significance of differences among the means. The data are presented as mean and standard deviation.

## 4. Conclusions

Thus, the study of the growth dynamics of *S. betulifolia* ssp. *aemiliana* and the biosynthesis of biologically active substances in a solid and liquid system in vitro revealed time points of maximum growth and biosynthetic activity of the culture. A similarity in growth trends and biosynthetic characteristics of *Spiraea* microshoots between the two systems was revealed. The highest productivity of culture in vitro was observed from the 35th to the 60th day of cultivation. Nonetheless, all parameters of growth and biosynthetic activity were greater in the liquid culture system. For example, the content of phenolcarboxylic acids and antiradical activity in *S. betulifolia* ssp. *aemiliana* cultivated in a liquid medium are higher than those in microshoots cultivated on a solid medium. A comparative phytochemical analysis of microshoots in vitro, intact plants, and ex vitro–acclimatized plants was also carried out. It was found that the concentration of kaempferol glycosides is higher in microshoots obtained in vitro than in intact plants and in ex vitro–acclimatized plants. Interest in the research on phenolcarboxylic acids and kaempferol glycosides is due to their high pharmacological and nutraceutical potential. The cultivation of *S. betulifolia* ssp. *aemiliana* in the liquid system in vitro showed promise for obtaining standardized medicinal raw materials rich in the above metabolites; therefore, the optimization of concentrations of mineral salts in the nutrient medium will be the subject of further research to achieve even greater productivity of microshoots.

## Figures and Tables

**Table 1:** Levels of flavonoids and phenol carboxylic acids in water-ethanol leaf extracts of *S. betulifolia* ssp. *aemiliana* introduced into the Central Siberian Botanical Garden (CSBG), the Siberian Branch of the Russian Academy of Sciences (SB RAS).

Group Name	Detailed Information (Date of Sampling)	Content of Substances, % of ADM	
		Flavonoids	Phenol Carboxylic Acids
IPs	Intact plants brought from Kunashir Island that have been introduced into the experimental field of CSBG SB RAS since 2016 (June 2021)	3.80 ± 0.31	6.47 ± 0.58
APs	Ex vitro–acclimated plants: plants adapted from in vitro culture and grown in open ground that have been introduced into the experimental field of CSBG SB RAS since 2020 (June 2021)	3.81 ± 0.34	7.50 ± 0.67

Note. ADM: absolutely dry mass.

**Table 2:** Characteristics and levels of the phenol carboxylic acids detected by HPLC in *S. betulifolia* ssp. *aemiliana* extracts.

Peak No.	Compound	Spectral Characteristics (λ_max_, nm)	Retention Time (t_R_), min	Content, mg/g of Absolutely Dry Mass			
				In Vitro		In Vivo	
				MshS	MshL	IPs	APs
Extracts (solvent system I)							
A1	chlorogenic acid	244, 300 sh., 330	3.2	0.32 ± 0.01 ^b^	0.46 ±0.01 ^a^	0.46 ± 0.01 ^a^	0.38 ± 0.02 ^b^
A2	*p*-coumaric acid	226, 293, 320	7.9	0.04 ± 0.02 ^c^	0.12 ± 0.03 ^c^	0.42 ± 0.04 ^b^	0.66 ± 0.04 ^a^
A3	cinnamic acid	216, 270	35.9	–	–	0.43 ± 0.08 ^a^	0.42 ± 0.05 ^a^
A4	phenolic acid 4	255, 265 sh., 315	43.1	0.75 ± 0.07 ^b^	0.73 ± 0.03 ^b^	2.76 ± 0.13 ^a^	2.77 ± 0.10 ^a^

Notes. Mean values and standard deviation (*n* = 3) are presented. Means with different superscript letters (a,b,c, or d) in the same row are significantly different (*p* ≤ 0.05) according to Tukey’s honestly significant difference (HSD) test; “–“: not detected; sh.: shoulder.

**Table 3:** Characteristics and levels of the flavonoids detected by HPLC in *S. betulifolia* ssp. *aemiliana* extracts.

Peak No.	Compound	Spectral Characteristics (λ_max_, nm)	Retention Time (t_R_), min	Content, mg/g of Absolutely Dry Mass			
				In Vitro		In Vivo	
				MshS	MshL	IPs	APs
Extracts (solvent system I)							
F1	taxifolin	290	8.5	–	0.12 ± 0.01 ^b^	0.50 ± 0.07 ^a^	0.65 ± 0.08 ^a^
F2	hyperoside	225, 268 sh., 355	18.0	–	–	1.38 ± 0,14 ^a^	1.59 ± 0,07 ^a^
F3	isoquercitrin	259, 266 sh., 358	19.3	–	–	0.42 ± 0.01 ^b^	0.81 ± 0.09 ^a^
F4	rutin	256, 358	20.0	–	–	0.28 ± 0.01 ^a^	0.33 ± 0.04 ^a^
F5	flavone 5	250, 340	23.8	–	0.06 ± 0.01 ^b^	1.09 ± 0.13 ^a^	0.83 ± 0.18 ^a^
F6	astragalin	265, 300 sh., 350	32.5	–	0.12 ± 0.01 ^b^	0.57 ± 0.09 ^a^	0.65 ± 0.07 ^a^
F7	quercetin	255, 372	40.6	0.13 ± 0.04 ^b^	0.18 ± 0.01 ^b^	2.55 ± 0.15 ^a^	2.98 ± 0.13 ^a^
F8	kaempferol	266, 370	46.9	0.31 ± 0.01 ^a^	0.25 ± 0.01 ^b^	0.12 ± 0.02 ^c^	0.14 ± 0.01 ^c^
Hydrolysates of extracts (solvent system II)							
Aglycone 1	Quercetin *	255, 372	6.4	1.38 ± 0.07 ^b^	1.24 ± 0.06 ^b^	9.23 ± 0.19 ^a^	8.83 ± 0.18 ^a^
Aglycone 2	Kaempferol *	266, 370	11.0	1.33 ± 0.04 ^a^	1.06 ± 0.08 ^b^	0.78 ± 0.04 ^c^	0.88 ± 0.06 ^b,c^

Notes. Mean values and standard deviation (*n* = 3) are presented. Means with different superscript letters (a,b,c, or d) in the same row are significantly different (*p* ≤ 0.05) according to Tukey’s HSD test; “–“: not detected; sh.: shoulder. * The concentrations of aglycones were converted to those of the corresponding glycoside via coefficients from the literature (Section 3.7).

## Data Availability

Not applicable.
